# P-286. Prevalence of Pseudomonas aeruginosa in patients with ICU-related respiratory Infections: A secondary analysis of the European Network for ICU-Related Respiratory Infections (ENIRRIs)

**DOI:** 10.1093/ofid/ofae631.489

**Published:** 2025-01-29

**Authors:** Cristian C Serrano-Mayorga, Juan Olivella-Gomez, Antoni Torres, Ignacio Martin-Loeches, Luis Felipe F Reyes

**Affiliations:** Universidad de La Sabana, Chía, Cundinamarca, Colombia; Clínica Universidad de La Sabana, Bogotá, Distrito Capital de Bogota, Colombia; Hospital Clinic of Barcelona, Barcelona, Catalonia, Spain; Saint James University Hospital, Dublin, Dublin, Ireland; Universidad de La Sabana, Chía, Cundinamarca, Colombia

## Abstract

**Background:**

Microorganisms causing healthcare-associated infections are usually difficult to treat Gram-negative bacteria, making the empiric antibiotic selection challenging. Pseudomonas aeruginosa (PA) is a microorganism associated with hospital-acquired infections, and its rate of antimicrobial resistance has increased in the last ten years. However, the international prevalence of P. aeruginosa in ICU-Related Respiratory Infections (ICU-RRI) is unknown. This study aims to bridge this gap in the literature using a multicenter cohort study.

**Figure 1:**
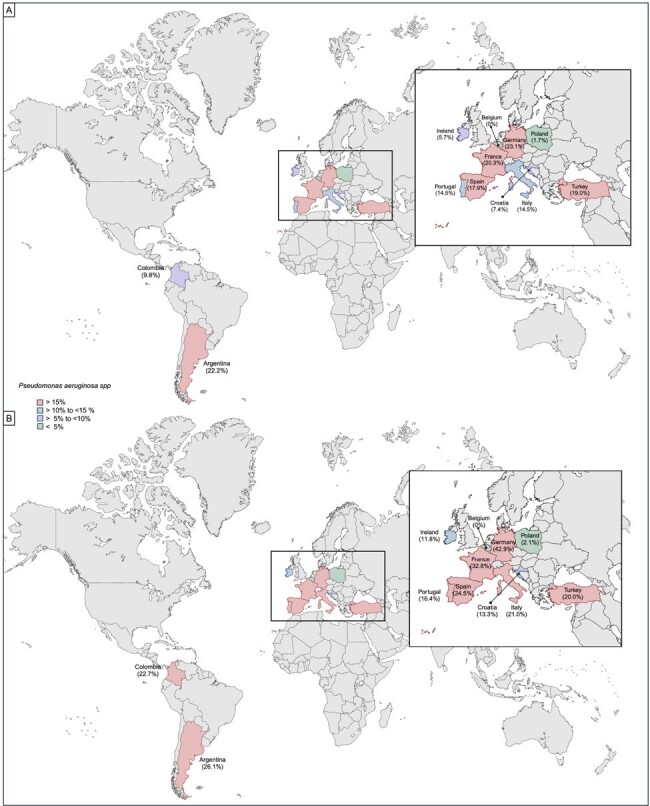
Prevalence of Pseudomonas aeruginosa infection by countries

Figure 1: Prevalence of Pseudomonas aeruginosa infection by countries for participants in the entire cohort (A) and bacterial microbiological Identification (B) Europe is shown in detail because of the high number of patients enrolled and the large number of participating countries.

**Methods:**

This prospective cohort was conducted in 12 countries over two continents from 9th May 2016 until 16th August 2019. Characteristics and outcomes of VAP, VAT, ICU-HAP, HAP (i.e., patients transferred to the ICU without requiring invasive mechanical ventilation), and HAP that required invasive ventilation (VHAP) were collected. All patients were tested to find microbiological agents and their resistance phenotype. We assessed the prevalence of Pseudomonas aeruginosa ICU-RRI.

**Figure 2:**
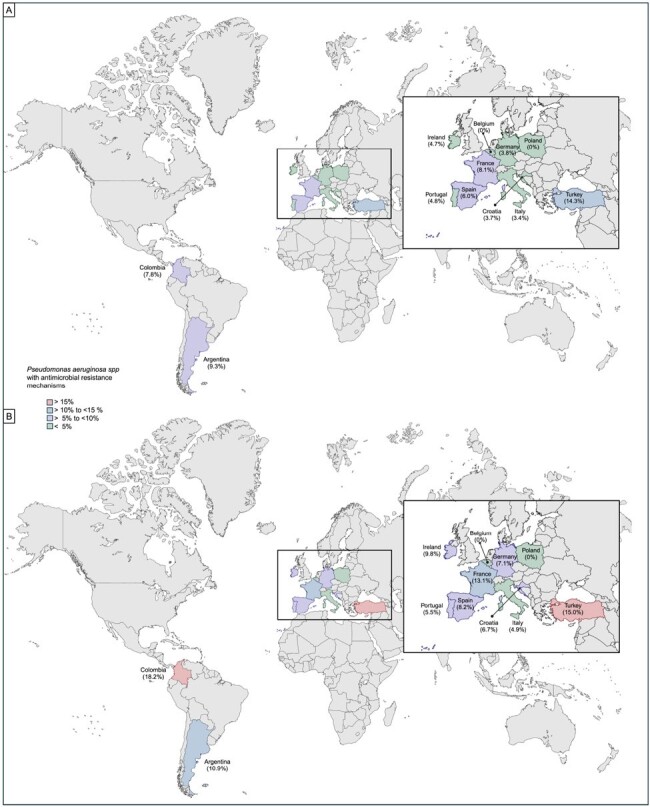
Prevalence of Pseudomonas aeruginosa with antimicrobial resistance infection by countries

Figure 2 Prevalence of Pseudomonas aeruginosa with antimicrobial resistance infection by countries for participants in the entire cohort (A) and bacterial microbiological Identification (B) Europe is shown in detail because of the high number of patients enrolled and the large number of participating countries.

**Results:**

1059 patients with ICU-RRI who underwent testing for microbiological diagnosis were included, and 710 (67.0%) had bacterial microbial identification. The overall prevalence of confirmed PA was 14.5% (n=153), and the overall prevalence of PA with at least one mechanism of antimicrobial resistance was 5,1% (n=55), with differing prevalence among the countries (Table 1/ Figure 1-2). 19.6 % (30/55) of the PA with at least one mechanism of antimicrobial resistance were catalogued as multidrug-resistant, and 11.1% (17/55) presented carbapenemase and beta-lactamase production (Table 2). Germany followed by Argentina and France were the countries with a major overall prevalence of PA. Turkey followed by Argentina and France were the countries with a major overall prevalence of PA with at least one mechanism of antimicrobial resistance (Figure 1-2).

**Table 1.**
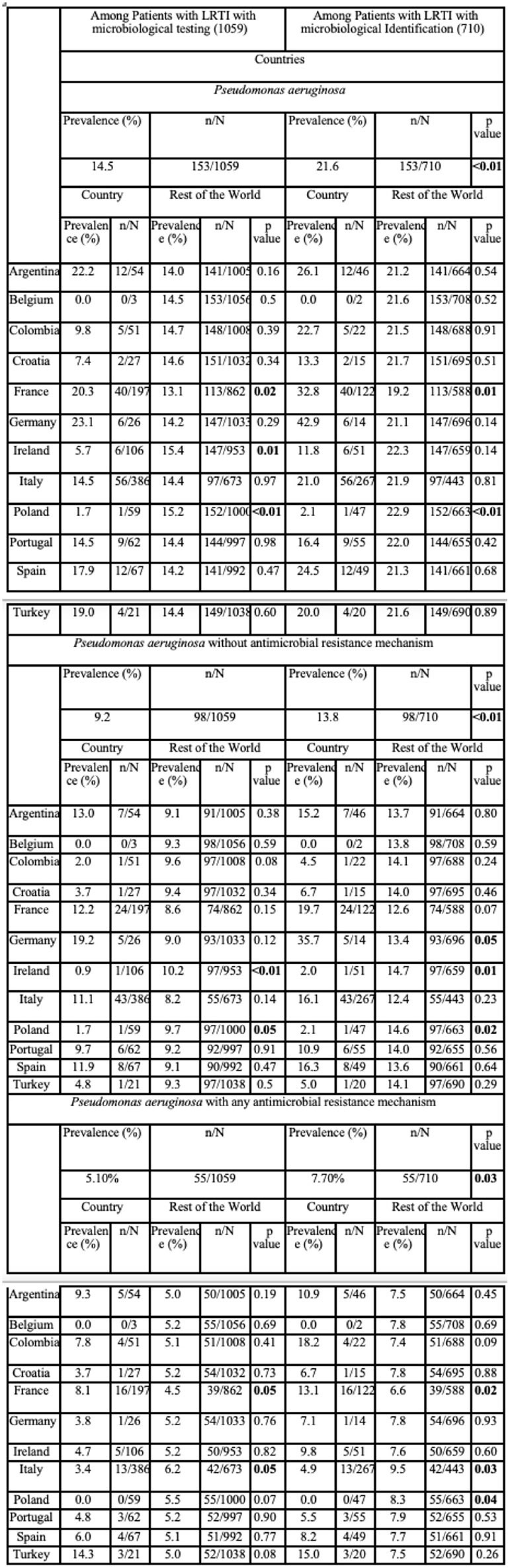
Prevalence of positive testing for Pseudomonas aeruginosa, Pseudomonas aeruginosa without antimicrobial resistance mechanism and Pseudomonas aeruginosa with any antimicrobial resistance mechanism among 12 countries in LRTI diagnosed patients who were admitted to the ICU.

**Conclusion:**

This multi-country study shows a high prevalence of PA and PA with at least one mechanism of antimicrobial resistance in ICU-RRI.

**Table 2.**
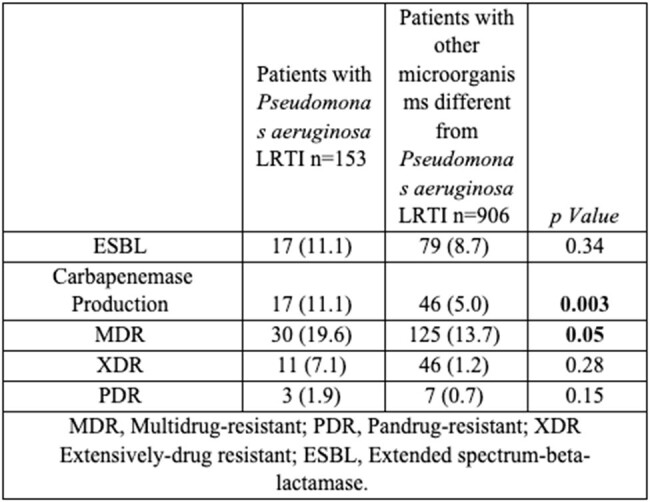
Description of antimicrobial resistance in LRTI-diagnosed patients who were admitted to the ICU.

**Disclosures:**

**All Authors**: No reported disclosures

